# Insight into the Mechanism of Action of Marine Cytotoxic Thiazinoquinones

**DOI:** 10.3390/md15110335

**Published:** 2017-11-02

**Authors:** Concetta Imperatore, Paola Cimino, Gerardo Cebrián-Torrejón, Marco Persico, Anna Aiello, Maria Senese, Caterina Fattorusso, Marialuisa Menna, Antonio Doménech-Carbó

**Affiliations:** 1 The NeaNat Group, Department of Pharmacy, University of Naples Federico II, Via D. Montesano 49, 80131 Napoli, Italy; cimperat@unina.it (C.I.); m.persico@unina.it (M.P.); aiello@unina.it (A.A.); maria.senese@unina.it (M.S.); 2 Italian Malaria Network—Centro Interuniversitario di Ricerche Sulla Malaria (CIRM), Dipartimento di Medicina Sperimentale e Scienze Biochimiche, via Del Giochetto, 06122 Perugia, Italy; 3 Department of Pharmacy, University of Salerno, Via Giovanni Paolo II 132, Fisciano, 84084 Salerno, Italy; cimino@unisa.it; 4 Departament de Química Analítica, Facultat de Química, Universitat de València, Dr. Moliner 50, Burjassot, 46100 Valencia, Spain; gerardo.cebrian_torrejon@unimes.fr; 5 Departement Des Sciences, Université de Nîmes University, Nimes EA7352 CHROME, Rue du Dr. G. Salan, 30021 Nîmes CEDEX 1, France

**Keywords:** bioactive natural products, thiazinoquinones, electrochemistry, DFT calculations, reactive radical species, cytotoxic activity

## Abstract

The electrochemical response of four natural cytotoxic thiazinoquinones isolated from the *Aplidium* species was studied using conventional solution-phase and solid-state techniques, based on the voltammetry of immobilized particles methodology. The interaction with O_2_ and electrochemically generated reactive oxygen species (ROS) was electrochemically monitored. At the same time, a molecular modeling study including density functional theory (DFT) calculations was performed in order to analyze the conformational and electronic properties of the natural thiazinoquinones, as well as those of their reduced intermediates. The obtained electrochemical and computational results were analyzed and correlated to cytotoxic activity of these compounds, highlighting some features possibly related to their mechanism of action.

## 1. Introduction

Since the early times of organic polarography [1], considerable efforts have been devoted to correlating electrochemical parameters with biochemical properties of natural products. Electrochemical data can provide testing of specific pharmacological activity and compound–biological substrate interactions [2], and can be used to mimic biological redox processes [3,4] and promote the in situ generation of redox-active species (typically, reactive oxygen species (ROS)) [2], being also applicable for testing the antimalarial activity of drugs acting via the hemozoin mechanism [5] or screening DNA types [6,7]. Moreover, the scope of available electrochemical methodologies can be expanded using the voltammetry of immobilized microparticles (VIMP), a solid-state technique developed by Scholz et al. [8] which provides analytical information on a wide variety of sparingly soluble compounds [9].

**Figure 1:**
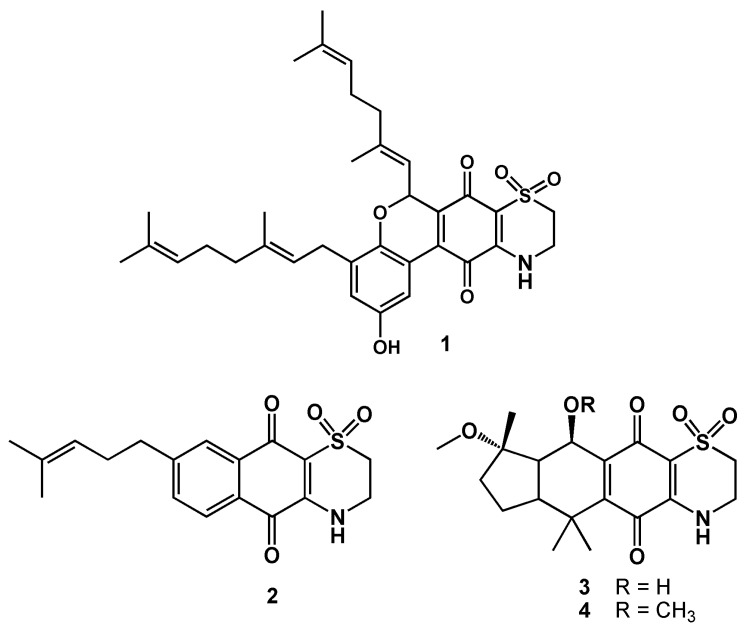
Structures of thiaplidiaquinone B (**1**); conicaquinone A (**2**); conithiaquinones A and B (**3**, **4**).

It is known that, because of their electrophilic properties, some natural quinones can react directly with cellular nucleophiles, including soluble and protein thiol groups, and may inhibit critical processes in the cell [10,11]. Some quinones intercalate between the base pairs of DNA leading to blockage of DNA, RNA, and protein synthesis [12], while some others stabilize binding of nuclear topoisomerase II to DNA resulting in protein-associated DNA strand breaks [13,14]. In addition, cytotoxic quinones can exert their effects through the formation of redox-reactive species [15,16,17]. Single-electron reduction of quinones is catalyzed by flavoenzymes, resulting in the formation of semiquinone free radicals. These free radicals can then either bind directly to DNA, protein, and lipid, or undergo further redox reactions [18,19,20,21]. In recent years, particular interest has been paid to quinone-based compounds forming reactive semiquinone free radicals which can then reduce oxygen to form reactive oxygen species (ROS), such as superoxide radicals (O_2_^•−^), and regenerate the parent molecule [15]. This pathway has been considered as operative for some natural quinones [22,23,24] but it is not the sole pathway leading to ROS generation. Semiquinone free radicals can indeed interfere with the whole electron transport chain in the mitochondria, being either oxidized or reduced [21]. In this scenario, thiaplidiaquinone B (**1**, Figure 1), a cytotoxic prenylated benzoquinone isolated from the marine ascidian *Aplidium conicum*, was proved [25] to induce apoptosis in Jurkat cells due to a rapid overproduction of intracellular ROS, which mediate the collapse of the mitochondrial transmembrane potential. Kinetic experiments showed that ROS production preceded the disruption of the mitochondrial potential and the appearance of apoptotic cells [25].

In the present work, VIMP was applied to the study of the electrochemistry of thiaplidiaquinone B (**1**) [25] as well as conicaquinone A (**2**), and conithiaquinones A and B (**3**, **4**), also isolated from *A. conicum* (Figure 1) [26,27].

The methodology previously used to study several synthetic quinone analogues [28] was expanded by combining conventional solution-phase and VIMP approaches incorporating electrochemically generated ROS [29,30,31] and by integrating computational studies performed on the starting quinone structures as well as on the reduced species.

## 2. Results and Discussion

### 2.1. General Voltammetric Pattern

**Figure 2:**
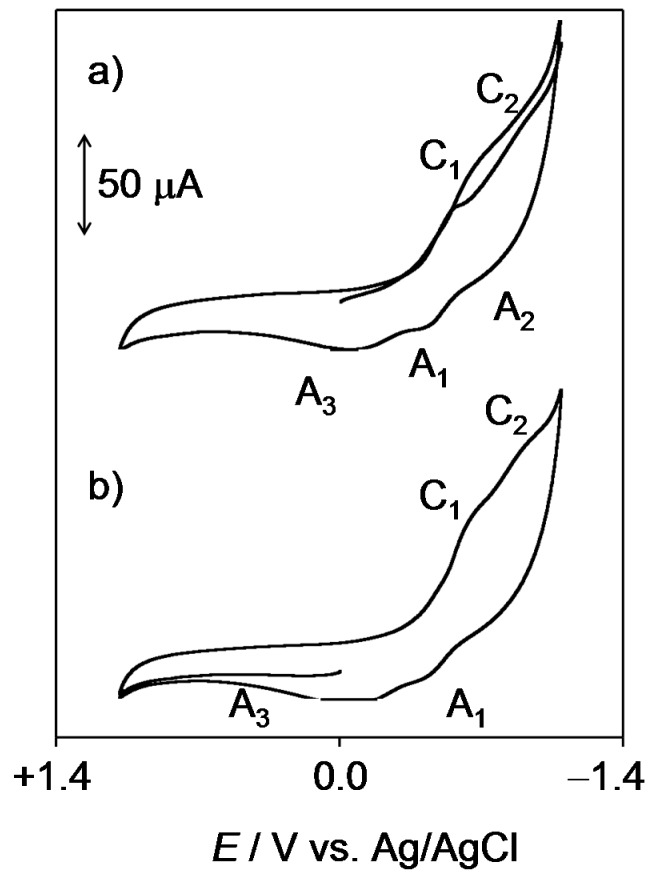
Cyclic voltammograms of **2** microparticulate films on a glassy carbon electrode immersed into 0.10 M PBS at pH 7.4. Potential scan initiated at 0.0 V (**a**) in the negative and (**b**) in the positive directions; potential scan rate 50 mV·s^−1^.

In view of the low solubility in water of the studied compounds, solid state electrochemistry was performed on microparticulate films of the compounds (**1**–**4**) immersed into aqueous PBS at physiological pH. Figure 2 shows the negative- and positive-going scan cyclic voltammograms of **2**.

**Figure 3:**
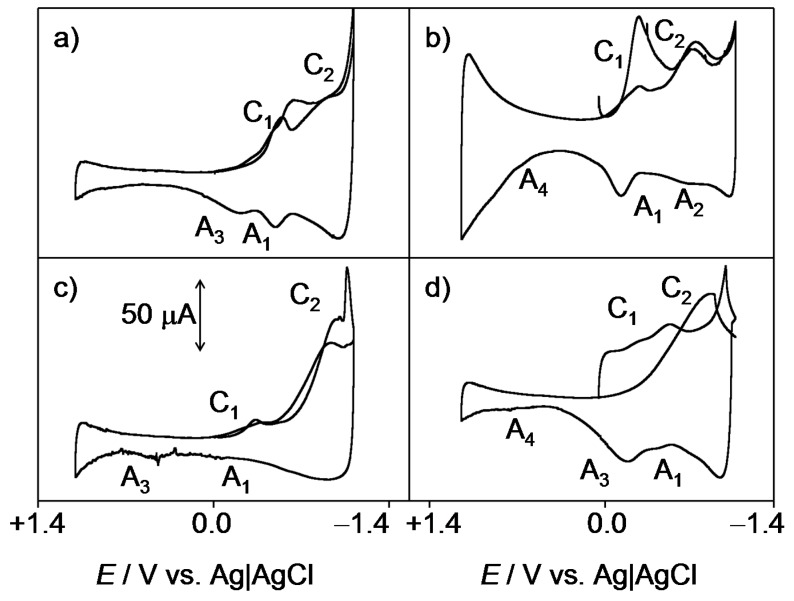
Cyclic voltammograms, after semi-derivative convolution, of (**a**) **2**; (**b**) **3**; (**c**) **4**; and (**d**) **1** microparticulate films on glassy carbon electrodes immersed into 0.10 M PBS at pH 7.4. Potential scan initiated at 0.0 V (**a**) in the negative and (**b**) in the positive directions; potential scan rate 50 mV·s^−1^.

In the initial cathodic scan (Figure 2a), reduction peaks appear at −0.65 V (C_1_) and −1.03 V (C_2_) vs. Ag/AgCl. These signals are coupled, in the subsequent anodic scan, with a weak shoulder at −0.80 V (A_2_) and a well-defined, although weak, oxidation peak at −0.45 V (A_1_) which precedes a broad oxidation signal at −0.04 V (A_3_). This peak is absent in initial anodic scan voltammograms (Figure 2b), thus denoting that the process A_3_ corresponds to the oxidation of any product generated in previous cathodic steps. The voltammetry of all the studied quinones was similar, however, the potential separation and the relative height of peaks C_1_ and C_2_ varied significantly. This can be seen in Figure 3, where semi-derivative deconvolution of the voltammetric curves was performed in order to enhance peak resolution. As can be seen in this figure, **2** and **3** display peaks C_1_ and C_2_ of comparable intensity and the peak A_1_ remains well-defined. In contrast, the peak C_1_ is relatively weak in **4** and **1** and the former exhibits weak signals A_1_ and A_3_, whereas the latter presents a relatively intense peak A_1_ without the accompanying peak A_3_.

**Scheme 1:**
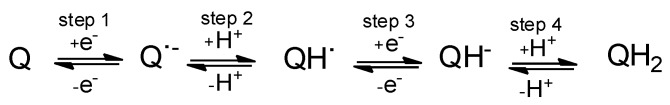
Reduction pathway for the electrochemical reduction of quinone to hydroquinone in aqueous environment.

This voltammetry can be rationalized on the basis of detailed knowledge of the electrochemistry of quinones in solution phase [32] and the Lovric and Scholz model on the electrochemistry of ion-insertion solids [33,34,35,36,37]. In the case of organic compounds in contact with aqueous electrolytes, solid-state electrochemical reactions are initiated at the solid particle/base electrode/electrolyte three-phase junction and propagates through the solid via proton insertion coupled with electron hopping between immobile redox centers in the solid [33,34,35,36,37,38]. The reduction of quinones (Q) to hydroquinones (QH_2_) in aqueous solution occurs typically via two rounds of electron-transfer (E) coupled with proton uptake (chemical reaction, C), i.e., ECEC mechanism. This reduction pathway produces the radical anion Q^●−^, the semiquinone radical QH^●^, and the anion QH^−^ species (Scheme 1).

The overall reduction process can be described in terms of an ECEC mechanism, in general producing two voltammetric peaks. Depending on the pH and the buffering capacity of the electrolyte, however, a unique voltammetric couple can be observed [1,33]. This situation operates in our case, where the cathodic wave of the C_1_/A_1_ couple often looks like two superimposed signals (Figure 3a,b).

As judged upon comparison with the electrochemistry of synthetic thiazinoquinones, [28] the above electrochemical process would be followed by the reduction of the SO_2_ unit to SO at more negative potentials (C_2_/A_2_ couple). The peak A_3_, which is depleted when the peak A_2_ is well developed (Figure 3b), can be attributed to the oxidation of the reduced form resulting from the initial reduction C_1_ through the NH motif. In some cases (Figure 3b,d), an ill-defined anodic shoulder ca. +0.6 V (A_4_) appears. This process can be assigned to the oxidation of the NH unit of the parent quinone [28].

### 2.2. Interaction with ROS

**Figure 4:**
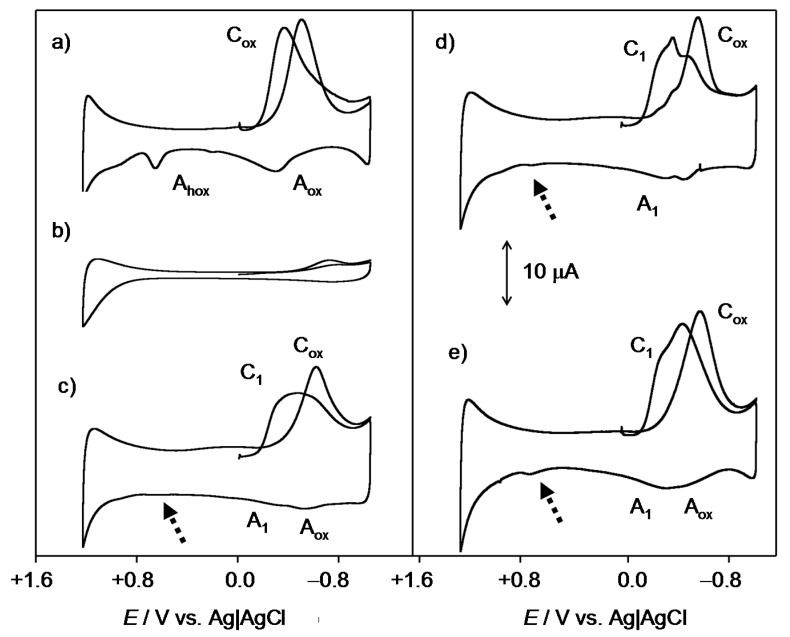
Cyclic voltammograms, after semi-derivative deconvolution, of an unmodified glassy carbon electrode in (**a**) air-saturated and (**b**) deoxygenated PBS solution at pH 7, and that electrode modified with (**c**) **3**; (**d**) **2**; and (**e**) **1** all in contact with air-saturated PBS. Potential scan rate 50 mV·s^−1^.

In order to test the possible interaction of the studied compounds with ROS, the voltammetry of microparticulate films of the same compounds in contact with air-saturated PBS was carried out. Figure 4 compares the cathodic response of an unmodified glassy carbon electrode with those of microparticulate films of the studied compounds on that electrode all in contact with air-saturated PBS at physiological pH of 7.4.

At the unmodified glassy carbon electrode, dissolved oxygen yields an apparently irreversible reduction wave at ca. −0.85 V (C_ox_). This process has been described in literature in terms of an initial one-electron reduction of O_2_ to the radical anion superoxide O_2_^•−^ which is rapidly protonated and reduced to H_2_O_2_ (Equations (1)–(6)) [39,40,41]. The multistep mechanism proposed for the process C_ox_ may be, however, complicated by adsorption processes, so that the first step can be represented as [38,39,40]

O_2_ + e^−^ → O_2_^•−^ (ads). (1)

According to literature [39,40,41], the generated radical anion undergoes protonation (2) followed by electrochemical reduction (3):

O_2_^•−^ (ads) + H_2_O → HO_2_^•^ (ads) + OH^−^, (2)

HO_2_^•^ (ads) + e^−^ → HO_2_^−^. (3)

Alternatively, it undergoes disproportionation:

2O_2_^•−^ (ads) + H_2_O → HO_2_^−^ + O_2_ + OH^−^, (4)

leading to the final generation of H_2_O_2_:

HO_2_^−^ + H_2_O → 2HO^•^ + OH^−^, (5)

HO^•^ + HO^•^ → H_2_O_2_. (6)

Through this series of processes, reactive radicals HO_2_^•^ and HO^•^ can be formed at the electrode surface, in agreement with literature on electrochemical generation [29,30,31,32], and redox potentials [42,43] of ROS. Accordingly, the process C_ox_ in Figure 4a corresponds to a multistep electrochemical reduction of dissolved O_2_ resulting in the generation of ROS, the signals A_ox_ and A_hox_ being attributable to the oxidation of O_2_^•−^ and HO_2_^•^, respectively [29,30,31,32,44]. These signals disappear upon deoxygenation of the electrolyte solution (see Figure 4b).

The C_ox_ peak shows a weak anodic counterpart (A_ox_) and is accompanied by an oxidation peak at +0.75 V (A_hox_). At films of the natural thiazinoquinones (Figure 4c–e), the signal C_ox_ is preceded by the first reduction process of the compound (C_1_), corresponding, in PBS, to the formation of HQ^•^, and the peak A_ox_ is depleted. The depletion of the signal is relatively large for **2** and **3** and much less intense for **4** and **1**. Interestingly, in the anodic scan, the oxidation peak at ca. +0.75 V (A_hox_), appearing in the voltammograms at the unmodified glassy carbon electrode (Figure 4a), is also considerably weakened (see dotted arrows in Figure 4b–d).

**Figure 5:**
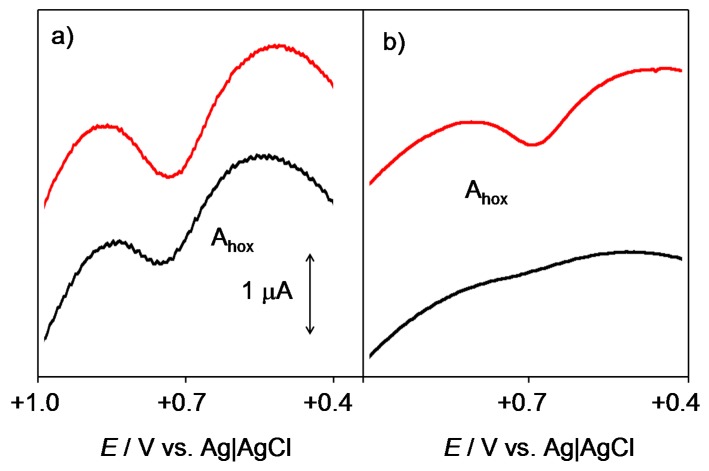
Square-wave voltammograms at unmodified (red lines) and **3**-modified (black lines) glassy carbon electrodes in contact with air-saturated PBS at pH 7.4. Potential scan initiated at −1.05 V in the positive direction; potential step increment 4 mV; square-wave amplitude 25 mV; frequencies of (**a**) 50 and (**b**) 5 Hz. Only the region of potentials around the peak A_ox_ is depicted.

The voltammetric features in Figure 4 can be rationalized by assuming that there is another pathway operating in the oxidation of dissolved peroxide/hydroperoxide at the quinone films, which competes with its electrochemical oxidation. This can consist of the reaction with the quinone. In order to test this possibility, high-frequency square-wave voltammetry was used to detect the presence of short-life intermediates, as described by Enache et al. [32]. Pertinent data for the hydroperoxide radical are presented in Figure 5, where the square-wave voltammograms of air-saturated PBS in contact with the unmodified and **3**-modified glassy carbon electrodes at two different square-wave frequencies are shown. At the unmodified electrode, the signal for HO_2_^•^ oxidation, A_hox_, appeared well defined at all frequencies, denoting the formation of this species during electrochemical runs. In contrast, at Q-modified electrodes, the signal for HO_2_^•^ oxidation was only recorded at relatively high frequencies thus suggesting that there is a relatively slow reaction with the Q film which consumes the generated HO_2_^•^ radical. Similar results were obtained for HO^•^, here producing an oxidation signal at ca. +1.2 V; pertinent data are provided as Supplementary information (Figure S3).

At a relatively high frequency (Figure 5a), i.e., at a short experimentation time, both the unmodified and **3**-modified electrodes display an essentially identical signal A_hox_, thus denoting that the generation of the hydroperoxide radical occurs. When the frequency is decreased below 10 Hz (Figure 5b), i.e., when the time is longer, the peak A_hox_ appears at the unmodified electrode whereas it vanishes at the **3**-modified electrode. It seems to be the most reasonable option to attribute the observed voltammetric features to the result of a relatively slow reaction of HQ^•^ with the electrochemically generated hydroperoxide. Accordingly, only when the time of experimentation is short, the signal of the unreacted hydroperoxide radical is recorded in the presence of **3**. This effect was observed to an essentially identical extent for the thiazinoquinones in this study, which is in agreement with the demonstrated ability of some quinones to modify the activity of superoxide dismutases (SOD) by undergoing equilibrium reactions with superoxide radicals [16,45].

**Figure 6:**
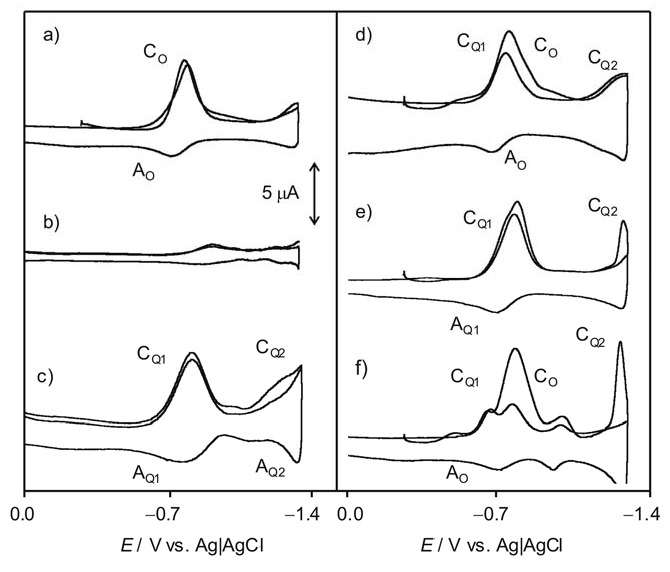
Cyclic voltammograms at a glassy carbon electrode, after semi-derivative convolution, of different 0.10 M Bu_4_NPF_6_/DMSO solutions. (**a**) air-saturated and (**b**) deoxygenated electrolyte solution; 0.5 mM **3** in (**c**) deoxygenated and (**d**) air-saturated electrolyte; (**c**) 0.5 mM **1** in (**e**) deoxygenated and (**f**) air-saturated electrolyte. Potential scan rate 50 mV·s^−1^.

Experiments in DMSO solution, also representative of the interaction of the studied compounds with oxygen and ROS, were used to complement the above scenario. Figure 6 compares the voltammograms of dissolved oxygen in 0.10 M Bu_4_NPF_6_/DMSO solutions (Figure 6a), **1** in the same electrolyte previously deoxygenated (Figure 6b) and air-saturated solutions of **3** (Figure 6c) and **4** (Figure 6d).

It is well known that the electrochemical reduction of dissolved oxygen in aprotic solvents occurs reversibly at potentials ca. −0.9 V (Figure 6a, C_O_/A_O_ couple) yielding a superoxide anion radical. In the absence of oxygen, the studied compounds produce an essentially reversible one-electron reduction (C_Q1_/A_Q1_) to the corresponding semiquinone at ca. −0.75 V followed by a second one-electron reduction at ca. −1.5 V (C_Q2_/A_Q2_). Voltammograms of air-saturated DMSO solutions of such compounds consist of superimposed signals for O_2_ and Q reduction but, remarkably, in the anodic scan, the anodic signal A_Q1_ is entirely or almost entirely depleted after passing the peak A_O_ which remains essentially unaltered, while the couple C_Q1_/A_Q1_ is also decreased.

The depletion of the peak A_Q1_ in the voltammograms of air-saturated thiazinoquinone solutions can be considered as indicative of the occurrence of a relatively fast reaction between dissolved oxygen and the semiquinone radical anion electrochemically generated. The large separation between the two one-electron reduction processes of the thiazinoquinones (see Figure 6b), in sharp contrast with the signal merging recorded in aqueous media (see Figure 2), denotes that there is no significant disproportionation of the intermediate semiquinone radical anion and there is no interaction of Q^•−^ with the superoxide anion. Thus, while the voltammetry of microparticulate films of the studied quinone in contact with air-saturated PBS revealed the reaction of the protonated semiquinone radicals by the superoxide anion radical, in air-saturated DMSO solution, on the contrary, the semiquinone anion radicals are oxidized by oxygen.

**Figure 7:**
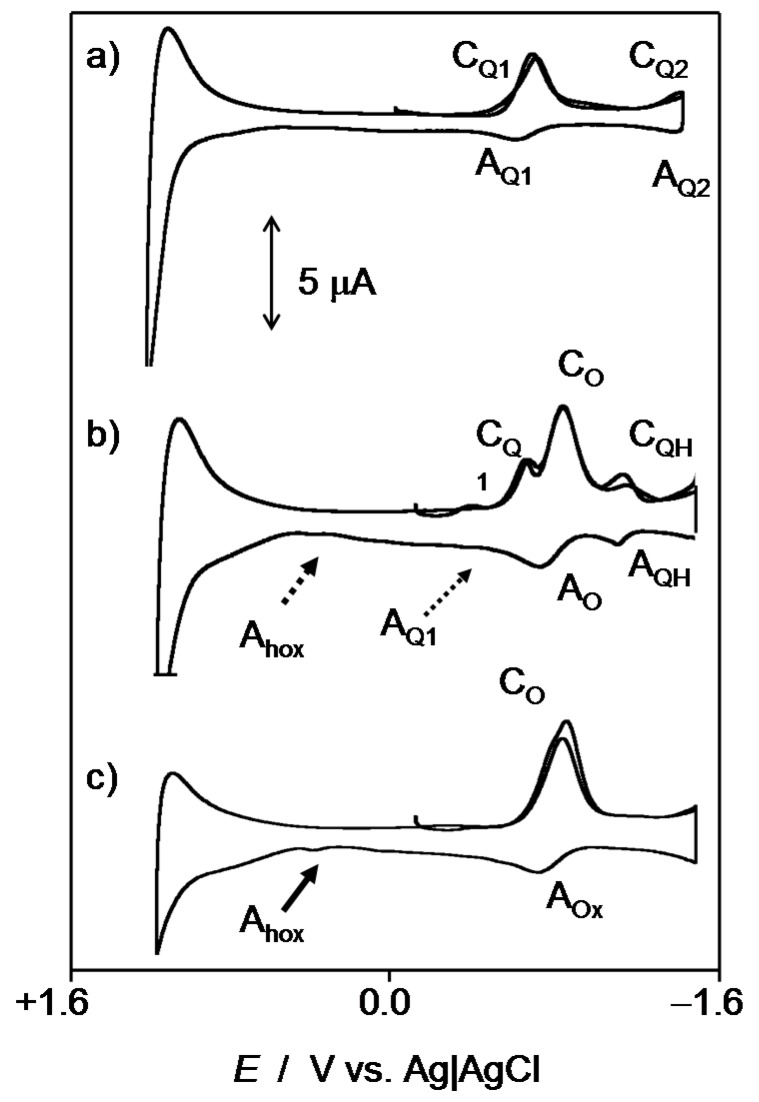
Cyclic voltammograms at a glassy carbon electrode, after semi-derivative convolution, of different 0.10 M Bu_4_NPF_6_/DMSO solutions. (**a**) 0.5 mM of **2**; (**b**) air-saturated 0.5 mM of **2** + 0.5 mM water; (**c**) air-saturated 0.10 M Bu_4_NPF_6_/DMSO +0.5 mM water. Potential scan rate 50 mV·s^−1^.

This difference can be assessed by performing additional experiments in water-containing DMSO solution. This can be seen in Figure 7 for **2**. In anhydrous DMSO in the absence of dissolved O_2_ (Figure 7a), the voltammetric response is identical to all other thazinoquinones in this study, with well-separated couples C_Q1_/A_Q1_ and C_Q2_/A_Q2_. In air-saturated DMSO containing a small concentration of water (Figure 7b), an additional couple (C_QH_/A_QH_) is recorded, corresponding to the overall reduction to hydroquinone, appearing as a unique voltammetric couple in the presence of water, that is, when the protonated semiquinone species can be formed (Figure 3). This new couple is placed at ca. 250 mV more negative potential than the C_Q1_/A_Q1_ couple corresponding to the reversible formation of Q^•−^. This new couple can be attributed to the reversible one-electron reduction of the protonated semiquinone resulting from the fast protonation of the semiquinone radical anion produced in the cathodic step C_Q1_. Consistently with this interpretation, even in a medium such as DMSO having high radical scavenging ability [16], a weak anodic signal (A_hox_) attributable to the formation of some radical hydroperoxide as a result of the presence of residual water, was recorded at the unmodified electrode (Figure 7c). Again, this signal vanishes more or less intensively in the presence of thiazinoquinones, now in the solution phase (Figure 7b).

In summary, the results of these voltammetric studies suggest that all the considered natural thiazinoquinones are able to form the semiquinone radical species which, depending on their protonation state, can be reduced or oxidized in presence of redox-active compounds, such as, in the case of these experiments, O_2_ and ROS.

### 2.3. Computational Studies

Computational studies were performed on the molecular models of compounds **1**–**4** (Figure 1) by using a combination of molecular dynamics (MD) and mechanic (MM) calculations with quantum-mechanical (QM) methods (PM7 and density functional theory (DFT)) (see the Materials and Methods section for details).

**Figure 8:**
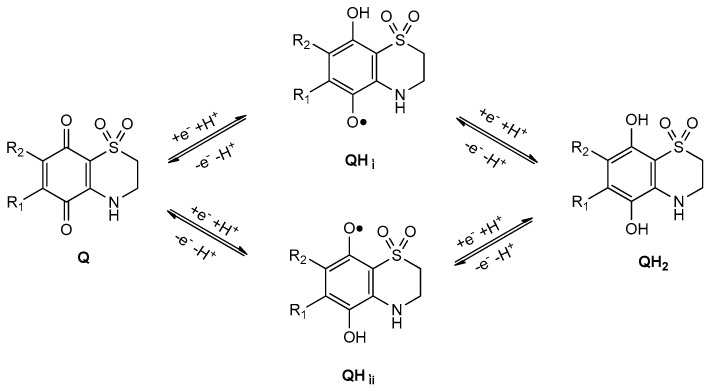
Schematic representation of the formation of the two possible protonated semiquinone radicals in the reduction pathway of natural thiazinoquinones.

The conformational and electronic properties of the compounds were investigated, taking into account the reduction pathway of quinones (Q) to hydroquinones (QH_2_) in an aqueous system**.** This involves a sequence of steps in which reversible one-electron reduction reactions are followed by proton uptake reactions producing different intermediate species, as illustrated in Scheme 1. In the case of the natural thiazinoquinones **1**–**4**, two possible protonated semiquinone radicals can be produced, i.e., QH^•^**_i_**, QH^•^**_ii_**, respectively (Figure 8).

**Figure 9:**
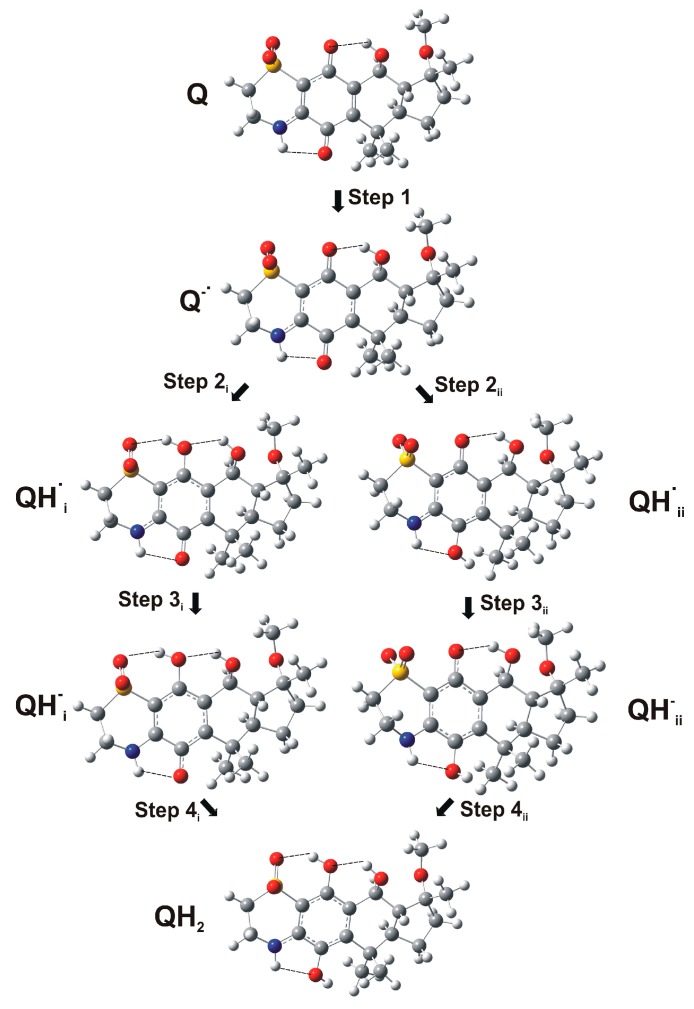
Q^•−^, QH^•^_i_, QH^•^_ii_, QH_i_^−^, QH_ii_^−^, and QH_2_ species of **3** obtained from DFT calculations.

Firstly, the conformational space of the starting neutral quinone species was sampled by applying a procedure including molecular dynamics (simulated annealing; SA), molecular mechanic (MM), and PM7 calculations. Obtained conformers were grouped into conformational families and ranked by their potential energy values. Secondly, the lowest energy conformer of each family was selected to be subjected to DFT full optimization. Finally, the DFT global minimum (GM) conformer of each compound was used to generate the reduced species Q^•−^, QH^•^_i_, QH^•^_ii_, QH^−^_i_, QH^−^_ii_, and QH_2_, which were again subjected to DFT full optimization (Figure 9).

In order to mimic an aqueous solution, all DFT calculations were performed using the conductor-like polarizable continuum model (C-PCM) [46] as the solvent model. Moreover, to characterize every structure as minimum and to calculate the Gibbs free energies, a vibrational analysis was carried out. This allowed the calculation of the Gibbs free energies of reaction Δ_r_*G*° = Σ(ε_0_ + *G*_corr_)_Prod._ − Σ(ε_0_ + *G*_corr_)_React._ of each step of the reduction pathway reported in Scheme 1. Due to the formation of the two possible semiquinone species QH^•^_i_ and QH^•^_ii_ (Figure 8), two different sets of reaction steps were calculated for the reduction from Q to QH_2_ (Table 1; Figure 9, Figures S1 and S2).

After the protonation of the radical anion Q^•−^, all compounds presented a hydrogen bond between the SO_2_ group and the newly generated hydroxyl group of QH^•^_i,_ which cannot be formed in the alternative QH^•^_ii_ semiquinone radical. This led to a more favorable Δ_r_*G*° for the formation of QH^•^_i_ with respect to that of QH^•^_ii_ (Step 2_i_ vs. Step 2_ii_; Table 1). On the other hand, the acquisition of a further electron, generating the corresponding QH^−^ radical anions, is thermodynamically more favored for the QH^•^_ii_ than for QH^•^_i_, regardless of the structural difference between the compounds (Step 3_i_ vs. Step 3_ii_; Table 1). It is noteworthy that, in any case, the larger contribution to the overall reaction energy comes from the protonation steps and this is in agreement with the role played by semiquinone radical anion protonation on quinone reduction, as evidenced by our electrochemical studies. Actually, many variables can affect the protonation rate of the reduced species, which can significantly differ in a cellular environment.

Some considerations can be made by comparing the results obtained for **3** and **4** with their relative potency in biological assay, since they were both tested on the same (MCF-7) cancer cell line [27]. Considering each reaction step (Scheme 1; Figure 8 and Figure 9), the more active compound **3** shows a higher propensity to acquire both the first and the second electron (Steps 1 and 3, respectively) with respect to **4** (Table 1; Figures S1 and S2). On the contrary, the two protonation steps (Step 2 and Step 4) present more favorable reaction energies for **4** with respect to **3**. Overall, according to the data reported in Table 2, the formation of the semiquinone species QH^•^_i_ is thermodynamically more favored for **3** (more active) than for **4** (less active).

To further investigate the redox properties of the studied bioactive quinones, we calculated their electrophilicity index (ω) [47] and vertical electron affinity (*EA*)Q, as well as the vertical *EA* and ionization potentials (*IP*) of the one-electron reduced forms Q^•−^ and QH^•^_i_. The vertical *EA* measures the propensity of a molecule to acquire one electron without considering subsequent electronic and/or structural rearrangements, which may drive the structure to a lower energy minimum (see the experimental section for details). Under the same conditions, the vertical IP measures the propensity of a molecule to lose one electron. The resulting ω, *EA*, and *IP* values are reported in Table 2, together with the energy of frontier orbitals of (i) the neutral quinone Q (LUMO); (ii) the anion radical Q^•−^ (SOMO); and (iii) the radical QH^•^_i_ (SOMO).

Following the same trend of the reaction energies reported in Table 1, compound **3** (more active) presents higher vertical *EA* and electrophilicity index and lower *E*_LUMO_ than those of **4** (less active). Moreover, the corresponding Q^•−^ and QH^•^**_i_** radicals of **3** present higher vertical *IP* and lower *E*_SOMO_ than those of **4** (Table 2). Similarly, the propensity of **1**—characterized by an IC_50_ of 3 μM on Jurkat cells—to acquire both the first and the second electron (Steps 1 and 3, respectively; Scheme 1; Table 1) is in line with the calculated LUMO energy, the vertical *EA*, and the electrophilicity index of the neutral form, as well as the SOMO energy and the vertical *EA* of the Q^•−^ radical form (Table 2). It is noteworthy that the higher SOMO energy and lower vertical IP of the semiquinone anion radical species compared to their protonated counterparts are in line with their different ability to react with redox-active compounds, such as O_2_ and ROS, as shown by the results of our voltammetry studies.

## 3. Materials and Methods

### 3.1. Reagents, Compound Isolation, and Characterization

Compounds **1**–**4** (Figure 1) were isolated and characterized as previously reported [25,26,27].

### 3.2. Electrochemical Studies

Electrochemical experiments were performed at 298 ± 1 K in a thermostated cell with CH I660 equipment (Cambria Scientific, Llwynhendy, Llanelli, UK). A BAS MF2012 glassy carbon working electrode (GCE) (geometrical area 0.071 cm^2^, BASi Corporate Headquarters, West Lafayette, IN, USA), a platinum wire auxiliary electrode, and an Ag/AgCl (3 M NaCl) reference electrode were used in a typical three-electrode arrangement. 0.10 M phosphate-buffered saline (PBS) at pH 7.4 (from Merck reagents, Darmstadt, Germany) was used for experiments in aqueous solution. Experiments in DMSO (Carlo Erba, Milan, Italy) were performed using 0.10 M Bu_4_NPF_6_ (Fluka, Munich, Germany) as a supporting electrolyte. Prior to electrochemical runs, all solutions were deaerated by bubbling Ar for a duration of 15 min.

The electrochemistry of films of the studied receptors on the glassy carbon electrode was studied using the methodology previously described [1] by pipetting 10 μL of a solution (1 mg/mL), previously ultrasonicated for 5 min, of the compound in ethanol and allowing the solvent to evaporate in air. As a result, a uniform, fine coating of the quinone was adhered to the basal electrode.

### 3.3. Computational Studies

Apparent p*K*_a_ values were calculated by using ACD/Percepta software [48]. The compounds were built, taking into account the prevalent ionic forms at physiological pH (7.4), using the Insight 2005 Builder module (Accelrys Software Inc., San Diego, CA, USA). Atomic potentials and charges were assigned using the CFF91 force field [49]. The conformational space of the compounds was sampled through 200 cycles of simulated annealing (*ε* = 80*r). The following protocol was applied: The system was heated up to 1000 K over 2000 fs (time step = 3.0 fs). The temperature of 1000 K was applied to the system for 2000 fs (time step = 3.0 fs) with the aim of surmounting torsional barriers. Successively, the temperature was linearly reduced to 300 K in 1000 fs (time step = 1.0 fs). The resulting conformations were then subjected to molecular mechanic (MM) energy minimization within an Insight 2005 Discover module (Accelrys Software Inc., San Diego, CA, USA) (CFF91 force field; *ε* = 80*r) until the maximum RMS derivative was less than 0.001 kcal/Å, using Conjugate Gradient [50] as the minimization algorithm. MM conformers were then subjected to a full geometry optimization by semi-empirical calculations, using the quantum mechanical method PM7 [51] in the Mopac2012 package [52] and EF (Eigenvector Following routine) [53] as the geometry optimization algorithm. The GNORM value was set to 0.01. To reach a full geometry optimization, the criteria for terminating all optimizations were increased by a factor of 100, using the keyword PRECISE. Resulting conformers were ranked by their potential energy values (i.e., Δ*E* from the global energy minimum) and grouped into conformational families. The occurrence rates and Δ*E*_GM_ range for each conformational family were calculated. The lowest energy minima of each family were then subjected to DFT calculations. All structures were fully optimized at the B3LYP/6-31 + G(d,p) level [54,55] using the conductor-like polarizable continuum model (C-PCM) [46]. The SCRF/CPCM method allows the calculation of the energy in the presence of a solvent. In this case, all structures were optimized as a solute in an aqueous solution. In order to characterize every structure as minimum and to calculate the Gibbs free energies of reaction, a vibrational analysis was carried out at the same level of theory, using the keyword freq. The RMS force criterion was set to 3 × 10^−4^ a.u. The calculations were carried out using the Gaussian 09 package [56].

Starting from the structure of the Q (i.e., the starting quinone species) GM conformers, the redox states Q^•−^, QH^•^_i_, QH^•^_ii,_ QH_i_^−^, QH_ii_^−^, QH_2_ were generated. In particular, following the reduction pathway of quinones reported in Scheme 1, each species was generated starting from the DFT-optimized species of the previous step. The Gibbs free energies of reaction (kcal/mol) for the electron/proton transfer of each step of the reduction reaction were calculated_._ In particular, Gibbs free energy calculations for electron attachment were accomplished by determining Δ*G* of the reaction Y + e^−^ → Y^−^ that is, Δ_r_*G*° (298.15 K)= Σ (ε_0_ + *G*_corr_)_Y−_ − Σ (ε_0_ + *G*_corr_)_Y_, where Y is the Q or QH^•^ DFT conformer. Gibbs free energies of reaction for proton attachment were calculated by determining Δ*G* of the reaction Y + H^+^ → YH that is Δ_r_*G*° = Σ (ε_0_ + *G*_corr_)_YH_ − Σ (ε_0_ + *G*_corr_)_Y_, where Y is Q^•−^ or QH^−^ DFT conformer [57].

For each considered redox state, the energy of the frontier molecular orbitals (HOMO, LUMO, and SOMO) were used to calculate the following parameters: (i) the electrophilicity index (ω); (ii) the vertical ionization potential (*IP*); and (iii) the vertical electron affinity (*EA*). The electrophilicity indices (ω) were calculated following the expression ω = (μ^2^/2η), where μ is the chemical potential given by μ = −(*IP* + *EA*)/2 and η is the chemical hardness given by η = (*IP* − *EA*) [47], where *IP* = *G*(*N* = *N*_0−1_) − *G*(*N* = *N*_0_) and *EA* = *G*(*N* = *N*_0_) − *G*(*N* = *N*_0+1_). Here, *N*_0_ is the number of electrons in the ground state of the (usually neutral) system. Thus, the single point Gibbs free energies (*G*) of the cationic (*N*_0−1_ electron system), and anionic (*N*_0+1_ electron system) forms for each considered species were calculated for each compound.

## 4. Conclusions

The electrochemical response of the natural thiazinoquinones **1**–**4** in aqueous environments, such as PBS, differs from that in polar non-aqueous solvents, such as DMSO, and such behavior affects their reaction with O_2_ and electrochemically generated ROS. Indeed, the whole of the voltammetric studies indicate that, upon one electron reduction, **1**–**4** form a semiquinone radical intermediate, which, depending on the considered solvent, can be reduced (when protonated) or oxidized (in the un-protonated form) by ROS and O_2_, respectively. In agreement with the electrochemical results, computational studies suggest that the cytotoxic activity of the natural thiazinoquinones may be related to their ability to undergo a one-electron reduction, as well as to the reactivity of the generated radical species according to their protonation state.

The whole of our studies demonstrates the potential of the use of electrochemical techniques, combined with DFT calculations, to gain information on bioactive compounds whose mechanism of action involves a redox reaction. In our case, the electrochemical response of thiazinoquinones **1**–**4**, interpreted in the light of computational studies, pointed out some redox features of natural thiazinoquinones possibly related to their cytotoxic mechanism of action.

## Figures and Tables

**Table 1:** Gibbs free energies of reaction (Δ_r_*G*°; kcal/mol) calculated for the proposed reduction pathway (Scheme 1) considering the formation of the two possible semiquinone species.

Compound	(Q → QH_2_)	Step 1	Step 2_i_	Step 2_ii_	Step 3_i_	Step 3_ii_	Step 4_i_	Step 4_ii_
**1**	−752.9	−98.0	−277.2	−267.3	−92.8	−99.3	−284.9	−288.3
**2**	−742.2	−90.6	−278.1	−270.8	−85.2	−95.9	−288.3	−284.9
**3**	−750.6	−96.6	−275.2	−268.8	−90.0	−100.4	−288.7	−284.7
**4**	−749.6	−93.1	−278.5	−271.4	−88.2	−95.4	−289.8	−289.7

**Table 2:** Calculated energy values (kcal/mol) of LUMO (*E*_LUMO_) and SOMO (*E*_SOMO_), electrophilicity index (ω), vertical ionization potentials (*IP*) and vertical electron affinity (*EA*) of the Q, Q^•−^, and QH^•^_i_ species of natural thiazinoquinones.

Compound	Q			Q^•^^−^		QH^•^_i_		
	*E*_LUMO_	Vertical *EA*	*ω*	*E*_SOMO_	Vertical *IP*	*E*_SOMO_	Vertical *IP*	Vertical *EA*
**1**	−88.234	90.73	141.84	−104.637	104.03	−125.038	123.56	87.35
**2**	−80.848	84.28	104.57	−97.716	97.18	−117.773	117.22	80.21
**3**	−86.923	89.55	113.48	−104.192	104.62	−125.678	126.18	83.93
**4**	−82.888	86.46	106.72	−100.220	101.05	−123.576	124.19	81.24

## Supplementary Materials

The following are available online at www.mdpi.com/1660-3397/15/11/335/s1, Figure S1: Gibbs free energies of reaction calculated for the proposed reduction pathway considering the formation of the semiquinone species QH^•^_i_; Figure S2: Gibbs free energies of reaction calculated for the proposed reduction pathway considering the formation of the semiquinone species QH^•^_ii_; Figure S3: square-wave voltammograms of an air-saturated PBS solution at pH 7.4 at unmodified and **1**-modified glassy carbon electrode.
