# Supplementary material for: Insight into the Mechanism of Action of Marine Cytotoxic Thiazinoquinones
Source: Mar Drugs. 2017 Nov 2;15(11):335. doi: 10.3390/md15110335 (PMC5706025; doi:10.3390/md15110335)
Supplement: Supplementary file 1 [file marinedrugs-15-00335-s001.doc]

**Supplementary materials for**

Insight into the mechanism of action of marine cytotoxic thiazinoquinones

Concetta Imperatore^1,2,§^, Paola Cimino^3,§^, Gerardo Cebrián-Torrejón^4,5,§^, Marco Persico^1,2^, Anna Aiello^1,2^, Maria Senese^1,2^, Caterina Fattorusso^1,2^,*, Marialuisa Menna^1,2^,* and Antonio Doménech-Carbó^4^,*

^1^ The NeaNat Group, Department of Pharmacy, University of Naples “Federico II”, Via D. Montesano 49, 80131 Napoli, Italy.; [cimperat@unina.it](mailto:cimperat@unina.it) (C.I:); [m.persico@unina.it](mailto:m.persico@unina.it) (M.P.); [aiello@unina.it](mailto:aiello@unina.it) (A.A.); [maria.senese@unina.it](mailto:maria.senese@unina.it) (M.S.)

^2^ Italian Malaria Network - Centro Interuniversitario di Ricerche Sulla Malaria (CIRM), Dipartimento di Medicina Sperimentale e Scienze Biochimiche, via Del Giochetto, Perugia, Italy

^3^ Department of Pharmacy, University of Salerno, Via Giovanni Paolo II 132, 84084 Fisciano, Salerno, Italy; [cimino@unisa.it](mailto:cimino@unisa.it) (P.C.)

^4^ Departament de Química Analítica, Facultat de Química, Universitat de València, Dr. Moliner 50, 46100 Burjassot, Valencia, Spain

^5^ Departement Des Sciences, Université de Nîmes Univ. Nimes, EA7352 CHROME, Rue du Dr G. Salan, 30021, Nîmes Cedex 1, France; gerardo.cebrian_torrejon@unimes.fr (G.C.T.)

***** Correspondence: [caterina.fattorusso@unina.it](mailto:caterina.fattorusso@unina.it) (C.F.), Tel.: +39-081-678-544; [mlmenna@unina.it](mailto:mlmenna@unina.it) (M.M.), Tel: +39-081-678-518; [antonio.domenech@uv.es](mailto:antonio.domenech@uv.es) (A.D.C.), Tel.: +34-963-543-157

^§^  These authors equally contributed to this work

TOC:

1. Figure S1 S2
2. Figure S2 S3
3. Figure S3 S4


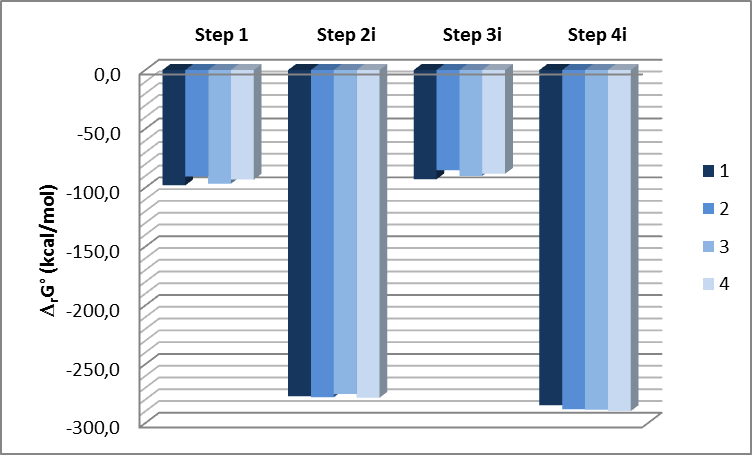


**Figure S1** Gibbs free energies of reaction (**Δ_r_G°;** kcal/mol) calculated for the proposed reduction pathway considering the formation of the semiquinone species QH^•^_i_.


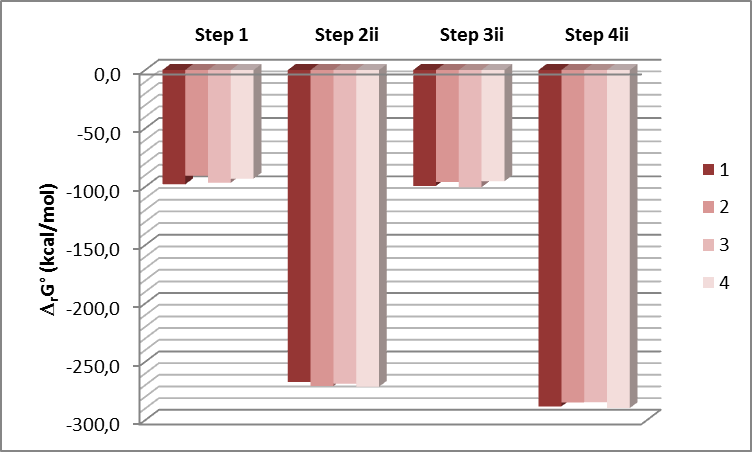


**Figure S2.** Gibbs free energies of reaction (**Δ_r_G°;** kcal/mol) calculated for the proposed reduction pathway considering the formation of the semiquinone species QH^•^_ii_.

**Figure S3.** Square wave voltammograms at an air-saturated PBS solution at pH 7.4 at a) unmodified and b) **1**-modified glassy carbon electrode. Potential scan initiated at -0.85 V in the positive direction; potential step increment 4 mV; square wave amplitude 25 mV; frequency 100 Hz. According to Enache et al. [32], the signal A_OH_ appearing at unmodified glassy carbon electrodes corresponds to the one-electron water oxidation yielding the HO^•^ radical. At thiazinoquinone-modified electrodes, this signal is depleted thus suggesting that there is a fast reaction with the Q-compound.
